# The Color-Word Stroop Task Does Not Differentiate Cognitive Inhibition Ability Among Esports Gamers of Varying Expertise

**DOI:** 10.3389/fpsyg.2019.02852

**Published:** 2019-12-20

**Authors:** Adam J. Toth, Magdalena Kowal, Mark J. Campbell

**Affiliations:** ^1^Lero Irish Software Research Centre, University of Limerick, Limerick, Ireland; ^2^Department of Physical Education and Sport Sciences, University of Limerick, Limerick, Ireland

**Keywords:** Stroop, counter-strike:global offensive, esport science, cognitive control, action video games, FPS

## Abstract

This study set out for the first time to identify whether gamers of low, intermediate, and elite skill level in a prominent esports game, Counter-Strike: Global Offensive, demonstrated increasingly superior performance on a test of a specific cognitive skill (cognitive inhibition). Here we tested low, intermediate, and high ranked gamers and compared their performance on a color-word Stroop Task and also compared the performance of players in each gaming rank group to non-gamers. Contrary to our hypothesis, the Stroop Task did not differentiate significantly gamers of varying expertise. Although, we found that when considering both accuracy and response times, elite gamers performed significantly better than both intermediate and low ranked gamers on the simple choice reaction time condition and both elite and novice gamers performed significantly better than intermediate ranked gamers on the incongruent condition (a measure of cognitive inhibitory ability).

## Introduction

Competitive video gaming, or esports (electronic sport), is a phenomenon that has grown dramatically over the past decade. From the development of professional gaming leagues, to the staggering numbers of spectators drawn to watching players compete, to the ever rising revenues every year, esports are solidifying their place in competitive sport culture (Wagner, 2006). Typically, sport involves the display of elite physical and cognitive skill in competition for entertainment purposes (Campbell et al., 2018). However, where traditional sports to a great extent rely on the development and performance of complex motor skills for success, gamers seem to rely more on cognitive skills (Himmelstein et al., 2017). As society continues to rely on digital technology for its entertainment, platforms such as twitch have revealed the immense popularity of watching elite gaming across the world. As we enter what may be the era of the cognitive athlete, the scientific investigation of esports must continue to grow.

Previous research on video gaming has debated numerous topics including the potential negative effects of action video gaming on behavior (Ferguson, 2007) and the effects of screen time on our physiology (Swing et al., 2010). However, a growing body of research has emerged demonstrating the benefits of video games for cognition. For example, a meta-analysis conducted by Bediou et al. (2018) demonstrated positive effects of gaming on cognitive abilities such as spatial memory, multi-tasking, and inhibition. Moreover, recent evidence has demonstrated that when compared to non-gamers, action video gamers display enhanced cognitive ability when evaluated using standardized cognitive measures of processing speed, visual search, and response inhibition (Kowal et al., 2018). However, despite the recent evidence in support of the effects of gaming on cognitive ability, no research to date has investigated whether superior cognitive ability is a hallmark of elite gaming performance.

Counter-Strike (now Counter-Strike:Global Offensive; CS:GO) is a first-person shooter computer game that has been one of biggest success stories for esports. Released in 1999, there have been a number of game releases prior to the current version and the game has been played professionally since 2012. In CS:GO, two teams of five players battle on a small map to either plant (terrorists) or diffuse (counter-terrorists) a bomb. Players are armed with weapons and while weapon proficiency is important, so are cognitive abilities like decision-making and response inhibition as friendly fire, an enabled feature of competitive CS:GO, makes recognizing the difference between friend and foe crucial for success. Despite the fact that anecdotally, elite players have a learned understanding of some of the important fundamentals to play CS:GO at a high level, gamers at all levels tend to practice very little on those specific abilities but rather, simply play more matches (Campbell et al., 2018). By better understanding the specific skills required for success in esports, players would be better able to comprehend the areas of strength and weakness in their performance, which has the potential to completely alter how esports athletes train and would align esports training with the type of training observed in traditional skill-based sports.

The purpose of this study is to identify whether gamers of low, intermediate, and elite skill level in a prominent esports game demonstrated increasingly superior performance on a test of a specific cognitive skill (cognitive inhibition). To address this purpose, we will evaluate the color-word Stroop performance (Stroop, 1935) among three groups of CS:GO gamers: those with low, intermediate, and elite level competitive game rankings. The Stroop Test is believed to measure a key cognitive inhibitory ability: response inhibition (MacLeod, 2007). Inhibition, traditionally considered as a critical component of executive functioning, is seen as the active suppression of task-irrelevant information from working memory, or the ability to inhibit an overlearned response (Strauss et al., 2006). We hypothesize that CS:GO gamers in higher ranking groups will demonstrate superior cognitive inhibitory ability compared to those in lower ranking groups evidenced by higher accuracy and faster response times, specifically on incongruent stimuli in the test.

## Methods

### Participants

One hundred and twenty-nine CS:GO players (*N* = 129; 126 males, 3 females) were recruited from attendees at the 2018 Gamescom and PAX gaming conference in Cologne, Germany, and Melbourne, Australia, respectively. Each provided informed consent prior to voluntarily participating in the study. The Research Ethics Board at the University of Limerick authorized approval for the study in accordance with the Declaration of Helsinki.

Participants began by completing a survey that gathered demographic information regarding their age, sex, handedness, and color vision. It also gathered data regarding their gameplay, including the average number of hours per week they estimated they spent playing CS:GO and their current competitive CS:GO ranking. Following completion of the survey, participants sat in front of a computer with a 24-inch monitor, were instructed to wear headphones to reduce the volume of external noise, and asked to complete a Color-Word Stroop Test.

### Stroop Test

**Figure 1:**
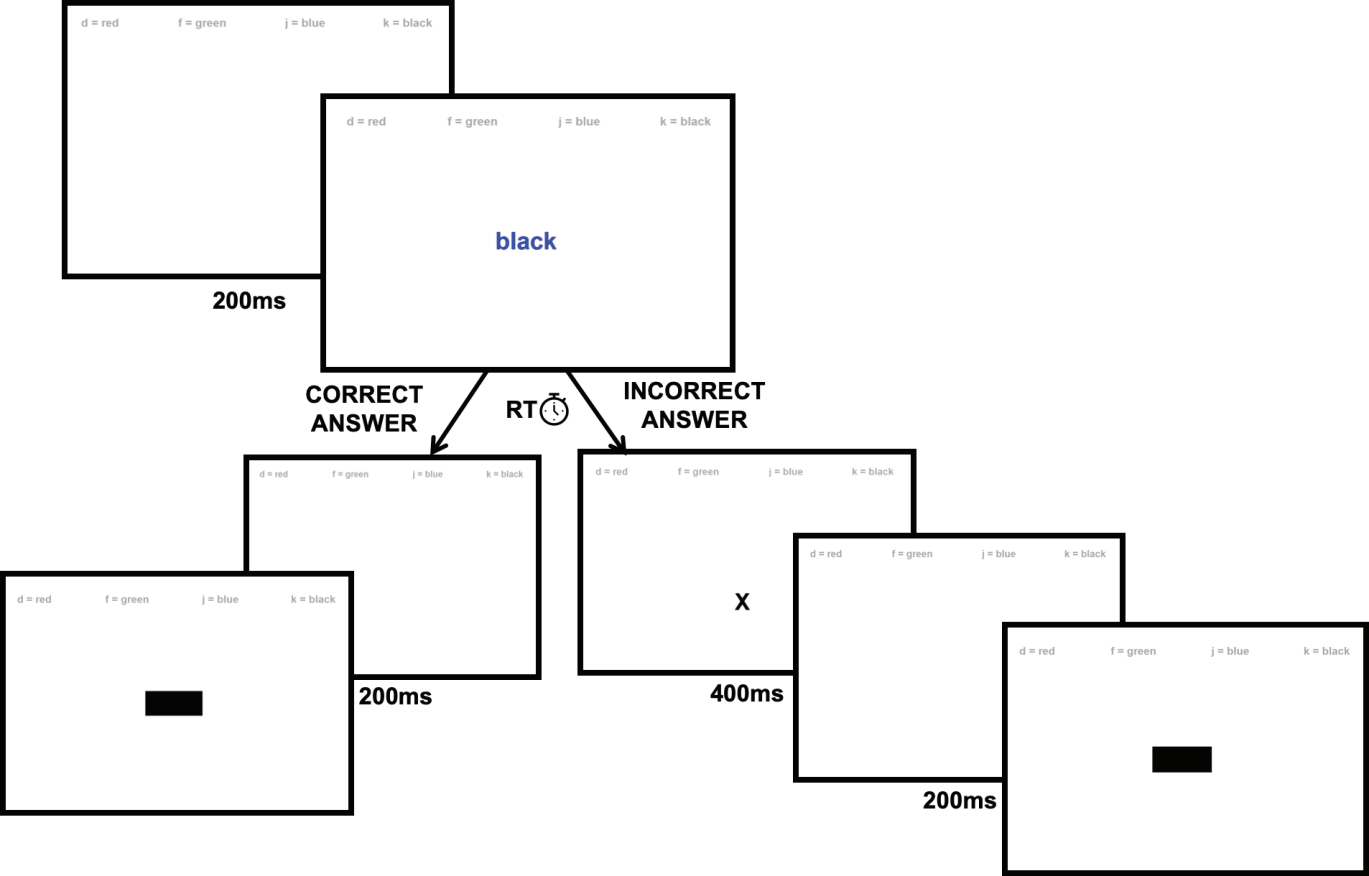
Example trial for the Color-Word Stroop Test. Participants are presented with a control, congruent, or incongruent trial after a brief 200-ms baseline (top left). If a participant responds correctly (left), the next trial is presented in identical fashion. If a participant responds incorrectly, a brief 400-ms × is shown on screen (right) to provide feedback prior to the presentation of the next trial.

The keyboard Color-Word Stroop Test used in this study was administered using Inquisit 4 software by Millisecond with the same test design used by Kowal et al. (2018). Participants were presented with either the word “red,” “green,” “black,” or “blue” on a white screen in either red, green, black, or blue colored font. In Congruent trials, the printed word and the color in which it was printed matched. Incongruent trials were those in which the printed word on screen and the font color it was printed in did not match. In addition to Congruent and Incongruent trials, Control trials were also included and consisted of a colored box presented on screen. In total, seven trials of each of the four colors within each condition (84 total trials) were presented randomly to participants during the test (see Figure 1). In every trial, participants were instructed to respond to the color of the ink used to present the word or box on screen and not the written word on screen. Participants were instructed to respond as quickly and accurately as they could using the keyboard keys “d,” “f,” “j,” and “k,” which corresponded with answers red, green, blue, and black, respectively. To aid participants, the key bindings were indicated in 18% neutral gray ink along the top of the screen throughout the test (see Figure 1). For each trial, the response was recorded as well as the reaction time (RT), in milliseconds, between the presentation of the stimulus and the participants’ response.

**Figure 2:**
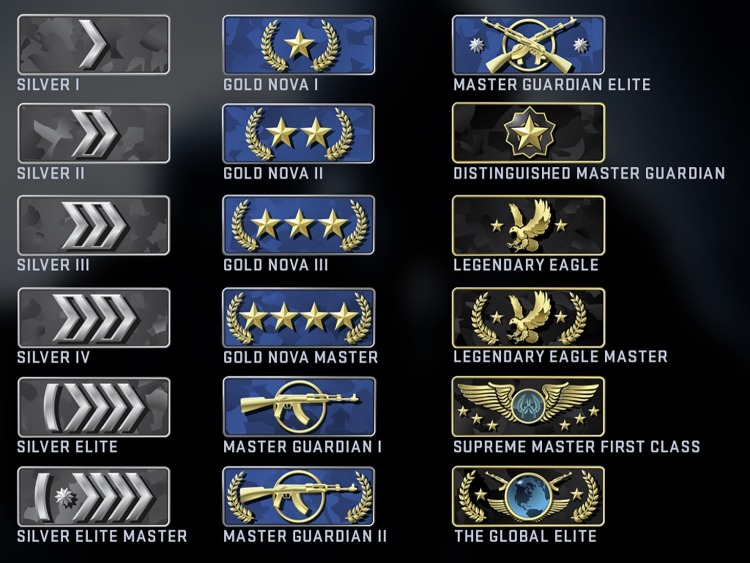
The 18 current competitive skill rankings for first-person shooter esports game, Counter-Strike:Global Offensive (CS:GO).

Participants’ data were excluded from analyses if they were under 16 or over 35 years of age (*n* = 7), indicated they were colorblind (*n* = 8) or were left-handed (*n* = 13). As evidence exists suggesting a female advantage on color-word Stroop Tasks (Golden, 1974) and due to our inability to compare male and female performance (low female *n*), we also excluded the data from the three female participants from further analyses. We additionally verified that no difference existed between our gamers from the Gamescom and PAX gaming conferences for Stroop accuracy [Site: *F*(1, 1,542) = 0.589, *p* = 0.443; Site*Condition: *F*(2, 1,542) = 1.434, *p* = 0.239] and RT [Site: *F*(1, 1,542) = 0.283, *p* = 0.595; Site*Condition: *F*(2, 1,542) = 0.010, *p* = 0.990] measures. The remaining participants were categorized based on their in-game competitive ranking. In total, there are 18 competitive CS:GO rankings (see Figure 2). We grouped participants based on their individual CS:GO ranking into one of three Rank groups. The Low Skill group consisted of gamers with rankings from Silver 1 to Silver Elite Master (*n* = 12, Age = 19.42 ± 3.44; Mean ± SD). The Intermediate Skill group contained gamers with rankings from Gold Nova 1 to Master Guardian 2 (*n* = 26, Age = 20.46 ± 4.16). Finally, the Elite Skill group consisted of gamers between the Master Guardian Elite to The Global Elite rankings (*n* = 60, Age = 19.77 ± 3.84).

Although previous work has determined that action video gamers display enhanced cognitive ability compared to non-gamers, this work never compared non-gamers to gamers of a specific game nor has it evaluated whether only gamers of a particular ranking showed superior performance. In order to address these questions, we also included Stroop performance data from a group of non-gamers published in a previous study (Kowal et al., 2018) and compared the performance of the 29 male participants in this group (Age = 21.28 ± 2.05) to the performance of ranked CS:GO players. Data for trials across each participant were also excluded if the response time (RT) for that trial exceeded that participant’s overall average by two standard deviations. The average (±SE) percent correct and average (±SE) response times are reported for each condition and each rank group.

### Statistical Analyses

In order to compare Stroop performance between the three CS:GO rank groups, we conducted two-way ANCOVAs on both Accuracy (Number Correct) and Reaction Time (RT; milliseconds) dependent variables with Condition (Control, Congruent, and Incongruent) and Rank group (non-gamers, Low Skill, Intermediate Skill, and Elite Skill) as independent variables. We also conducted one-way ANCOVAs on Stroop Interference Accuracy and Reaction Time dependent variables with Rank Group as an independent variable. Here, Stroop Interference is calculated as the average difference score between color-matched Incongruent and Control stimuli. Previous work has demonstrated superior cognitive performance with greater time allocated to gaming (Kowal et al., 2018). Therefore, the average number of hours reported gaming per week by participants was used as a covariate in the ANCOVAs. *Post hoc* analyses were performed with Tukey’s correction for multiple comparisons and significance was determined at an alpha level of 0.05. Descriptive metrics for rank groups and Stroop conditions are presented in Table 1.

**Table 1:** Accuracy (number correct) and response time (RT) descriptive metrics for rank groups and Stroop Task conditions.

Rank group	Condition	Number correct	SD	SE	95% CI	RT	SD	SE	95% CI
Non-gamers	Congruent	6.80	0.49	0.08	6.65, 6.95	884.42	247.79	23.62	838.12, 930.72
	Control	6.86	0.38	0.08	6.71, 7.01	864.50	239.41	23.73	818.00, 911.00
	Incongruent	6.67	0.59	0.08	6.51, 6.83	941.79	232.53	25.28	892.24, 991.34
Low skill	Congruent	6.53	0.72	0.11	6.32, 6.74	730.55	240.60	32.23	667.38, 793.72
	Control	6.32	0.84	0.11	6.11, 6.53	748.67	230.80	32.24	685.49, 811.85
	Incongruent	6.36	0.93	0.11	6.15, 6.57	814.84	229.00	32.94	750.27, 879.41
Intermediate skill	Congruent	6.72	0.60	0.07	6.58, 6.86	789.35	216.01	21.65	746.92, 831.78
	Control	6.65	0.62	0.07	6.51, 6.79	785.38	216.46	21.34	743.55, 827.21
	Incongruent	6.05	1.26	0.07	5.90, 6.20	889.21	248.67	22.53	845.04, 933.38
Elite skill	Congruent	6.64	0.67	0.05	6.55, 6.73	719.95	214.48	14.36	691.80, 748.10
	Control	6.58	0.69	0.05	6.49, 6.67	727.02	190.82	14.37	698.85, 755.19
	Incongruent	6.40	0.82	0.05	6.30, 6.50	784.59	218.02	14.89	755.41, 813.77

## Results

### Response Accuracy

**Figure 3:**
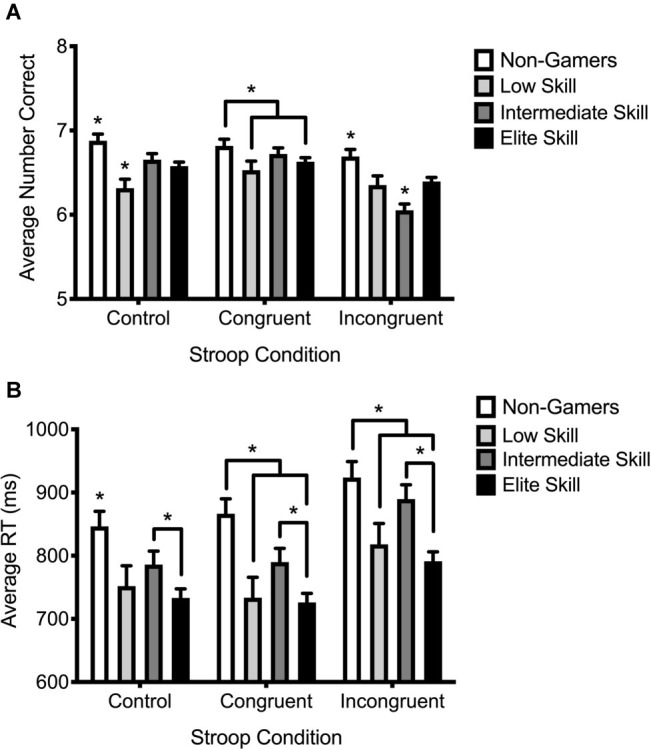
Average number of correct responses **(A)** and average response latencies in milliseconds **(B)** for Non-Gamer (white bars) and Low ranked (silver bars), Intermediate ranked (charcoal bars), and Elite ranked (black bars) CS:GO gamers across Control, Congruent, and Incongruent Stroop conditions. Error bars represent ±SE. ^*^indicates significant differences from all other bars unless asterisk brackets indicate otherwise.

Participants responded with accuracies of 94.4, 95.3 and 91.0% on Control, Congruent, and Incongruent trials respectively with response accuracy on incongruent trials being significantly poorer compared to those on control (*p* < 0.001) and congruent trials (*p* < 0.001). Although there were significant main effects for both rank group [*F*(3, 1,434) = 10.298, *p* < 0.001, *η*^2^ = 0.021] and condition [*F*(2, 1,434) = 15.823, *p* < 0.001, *η*^2^ = 0.022], there was a significant interaction between condition and rank group on response accuracy when controlling for the average hours per week participants gamed for [*F*(6, 1,434) = 3.918, *p* = 0.001, *η*^2^ = 0.016]. *Post hoc* comparisons revealed that non-gamers were significantly more accurate than CS:GO gamers across all Stroop conditions (Figure 3). Also, while no difference in accuracy was found between CS:GO rank groups for Congruent trials, Intermediate (*p* = 0.008) and Elite (*p* = 0.025) ranked CS:GO players were significantly more accurate than Low ranked gamers in the Control condition. In the Incongruent condition, Elite ranked gamers were significantly more accurate compared to intermediate ranked gamers (*p* < 0.001) but not Low ranked gamers (*p* = 0.723) (Figure 3). A significant main effect for Rank group was also found when analyzing Stroop Interference [*F*(3, 523) = 4.057, *p* = 0.038, *η*^2^ = 0.016] (see Supplementary Figure S1).

### Response Times

A significant main effect of condition [*F*(2, 1,443) = 13.49, *p* < 0.001, *η*^2^ = 0.018] was found and *post hoc* comparisons demonstrated that although participants responded to congruent and control trials with average latencies of 778.939 and 779.364 ms, respectively, they took significantly longer to respond to Incongruent trials (855.683 ms; *p* < 0.001). There was also a main effect of rank group [*F*(3, 1,442) = 17.962, *p* < 0.001, *η*^2^ = 0.036] whereby non-gamers were significantly slower than gamers in all CS:GO rank groups (Low; *p* < 0.001, Intermediate; *p* = 0.027, Elite; *p* < 0.001) and Elite ranked gamers showed significantly faster response times compared to Intermediate ranked gamers (*p* < 0.001). No interaction was found between condition and rank group [*F*(6, 1,434) = 0.382, *p* = 0.891, *η*^2^ = 0.002]. Finally, no effect for Rank group was found for Stroop Interference [*F*(3, 523) = 1.070, *p* = 0.361, *η*^2^ = 0.006] (see Supplementary Figure S1).

## Discussion

This study set out to identify whether gamers of low, intermediate, and elite skill level in a prominent esports game demonstrated increasingly superior performance on a test of a specific cognitive skill, previously suggested to be relevant for gaming performance. To do this, we tested gamers of the FPS game, CS:GO, on a standardized Color-Word Stroop Test and found evidence that elite ranked gamers show superior cognitive ability compared to lower ranked gamers. Specifically, we found that elite gamers have higher accuracy and faster response times for simple choice reaction time stimuli (control trials). However, contrary to our hypothesis, Stroop performance did not appear to differentiate cognitive inhibition among our three rank groups. Specifically, low and elite skill gamers performed better on incongruent stimuli compared to intermediate skilled gamers. These findings were also apparent when observing Stroop Interference measures. Finally, we corroborate previous work by demonstrating that gamers of all rankings prioritize speed over accuracy as a strategy when performing the Stroop Task (Kowal et al., 2018).

Traditional sport science, motor learning, and neuro-psychology research have demonstrated the importance of identifying and training the individual fundamental skills required for high performance (Mané et al., 1989; Boot et al., 2010; Conte et al., 2016). It is through the identification of an individual’s competency with the many physical and cognitive skills required for elite sports performance that tailored training plans can be developed to more rapidly improve performance. In fact, this has been shown using a game developed for psychological research called Space Fortress (Boot et al., 2010). Previous research used this game to show that those players who practiced the individual skills of the game in isolation improved their in-game performance significantly faster and to a greater extent compared to those who spent their training time simply playing the game. In addition to improving individual performance, knowledge of the unique combination of strengths and weaknesses across skills for all players of a team allows for the development of superior strategies that utilize players to maximize strengths and mitigate the effects of weaknesses during matches. Currently, very little research to date has attempted to identify the crucial cognitive and physical skills associated with elite esports performance. Moreover, competitive players often cite their practice regimen to involve a steady diet of matches or scrimmages to improve performance with little to no objective approach to training the fundamental skills required for high performance at their chosen game (Hollist, 2015). As esports performance research grows and competitive franchises begin to identify player skillsets and alter training strategies to improve performance, we may observe a significant evolution in esports and the quality of play required to compete at a high level.

In this study, we focused on the cognitive ability of cognitive inhibition, which has been identified as a skill superiorly displayed by action video gamers when compared to non-gamers. However, previous research has often combined gamers of different action video game genres. Previously, Campbell et al. (2018) suggested that esports games or genres should be viewed separately from one another, similar to the differentiation of different traditional sports. Here, we examined the performance on a well-known and widely utilized cognitive inhibition task among a homogeneous group of gamers of the FPS game CS:GO and compared their performance to a non-gaming sample. The apparent importance of cognitive inhibition for CS:GO and first-person shooter games in general may be tied to the importance of this cognitive skill for military personnel (Irgens-Hansen et al., 2015; Makhani et al., 2015). The scenario where distinguishing between friend and foe and deciding quickly and accurately whether to engage a target occurs regularly during CS:GO matches and often has a significant consequence to the outcome of a match.

This research is the first to attempt to quantify the influence that an individual’s cognitive skill has on differentiating players of different expertise level in a prominent esport. However, many more cognitive and physical abilities are likely to be important indicators of performance, and, by identifying the key skills and attributes that differentiate esports players of varying expertise, we may better understand how to develop training programs and in-game strategies to improve the probability of success for these individuals. To determine additional cognitive abilities associated with esports expertise, we may look to previous research that has identified specific cognitive abilities that are enhanced through gaming or which gamers show superiority with compared to non-gamers. For example, the meta-analytic work by Bediou and colleagues found that gamers were superior to non-gamers in the cognitive domains of inhibition, verbal cognition, perception, top-down attention, and spatial cognition (Bediou et al., 2018). These findings are supported by experimental work showing gamers possess enhanced spatial memory (Clemenson and Stark, 2015; Bonny et al., 2016) as well as visual attention and processing speed (Kowal et al., 2018) compared to non-gamers, but also that some of these cognitive aspects can be improved by gaming (Green et al., 2010; Boot et al., 2011; Green and Bavelier, 2012).

In addition to the cognitive skills that may mark performance, there remains a gap in esports performance science highlighting the physical skills and attributes that highlight expertise within different games. For example, it has been well established that elite players of the game *Starcraft* possess a unique ability to output a significantly higher number of actions per minute compared to low ranked players and non-gamers (Hotz, 2012). In CS:GO, players have highlighted skills such as “flicking” and “tracking” to be key mouse control skills allowing players to hit and kill targets with the greatest speed and efficiency. However, no research into the biomechanical and motor control skills displayed by elite esports players has been conducted to date and the area would immensely benefit from experiments that aim to quantify the magnitude of effect that different physical skills have on gaming performance.

Interestingly, among our sample of CS:GO players, we did not see that performance on the Stroop Task was better among individuals strictly in the higher ranking groups. Specifically, elite ranked gamers significantly outperformed intermediate ranked gamers but not those in the lowest rank group on incongruent stimuli. This may be due to the fact that the Stroop Task does not have the sensitivity to differentiate cognitive inhibitory ability among a homogeneous group of esports gamers or quite simply there were no cognitive inhibition differences in the first place among the groups. We encourage future work to investigate more aspects of cognition among esports players. Alternatively, the observed finding may be due to a differential influence that the many esports skills have on one’s performance as they gain expertise in the game. For example, a lack of expertise across a number of mechanical skills using their mouse and keyboard may more strongly differentiate low from intermediate ranked players. In this way, low ranked players may be more largely differentiated during gameplay by their physical, rather than cognitive, ability. As mechanical skill develops and becomes less influential on overall ranking differences among intermediate players, perhaps cognitive abilities, such as cognitive inhibition, become more important and thus the main obstacle for those unable to achieve an elite ranking status. In order to address this hypothesis, we recommend future research to identify the likely many more cognitive and physical markers of esports expertise, particularly in FPS games, and establish the other skills that differentiate specifically low ranked gamers and those in both intermediate and higher rankings.

This study tested whether cognitive inhibition, the mind’s ability to disregard stimuli that are irrelevant to the task at hand, and a known attribute of successful action video gaming could differentiate expertise among players of one of the most popular first-person shooter esports, Counter-Strike: Global Offensive. We encourage future research to continue toward the identification of the outstanding skills and characteristics underlying optimal esports performance. It is our hope that the current study helps to accelerate a new and emerging body of esports performance research that aims to revolutionize the methods used by gamers to train and prepare for elite competitive esports competitions.

## Data Availability Statement

The datasets generated for this study are available on request to the corresponding author.

## Ethics Statement

The studies involving human participants were reviewed and approved by Ethics Committee of the Faculty of Education and Health Sciences (EHSREC), University of Limerick. The patients/participants provided their written informed consent to participate in this study.

## Author Contributions

MC and AT conceptualized and drafted the article and revised it critically for important intellectual content; gave final approval of the version to be published; and were accountable for all aspects of the work. MK conceptualized and revised the study critically for important intellectual content, gave final approval of the version to be published, and was accountable for all aspects of the work.

### Conflict of Interest

The authors declare that the research was conducted in the absence of any commercial or financial relationships that could be construed as a potential conflict of interest.
