# Supplementary figures and images for: The Color-Word Stroop Task Does Not Differentiate Cognitive Inhibition Ability Among Esports Gamers of Varying Expertise
Source: Front Psychol. 2019 Dec 20;10:2852. doi: 10.3389/fpsyg.2019.02852 (PMC6932966; doi:10.3389/fpsyg.2019.02852)

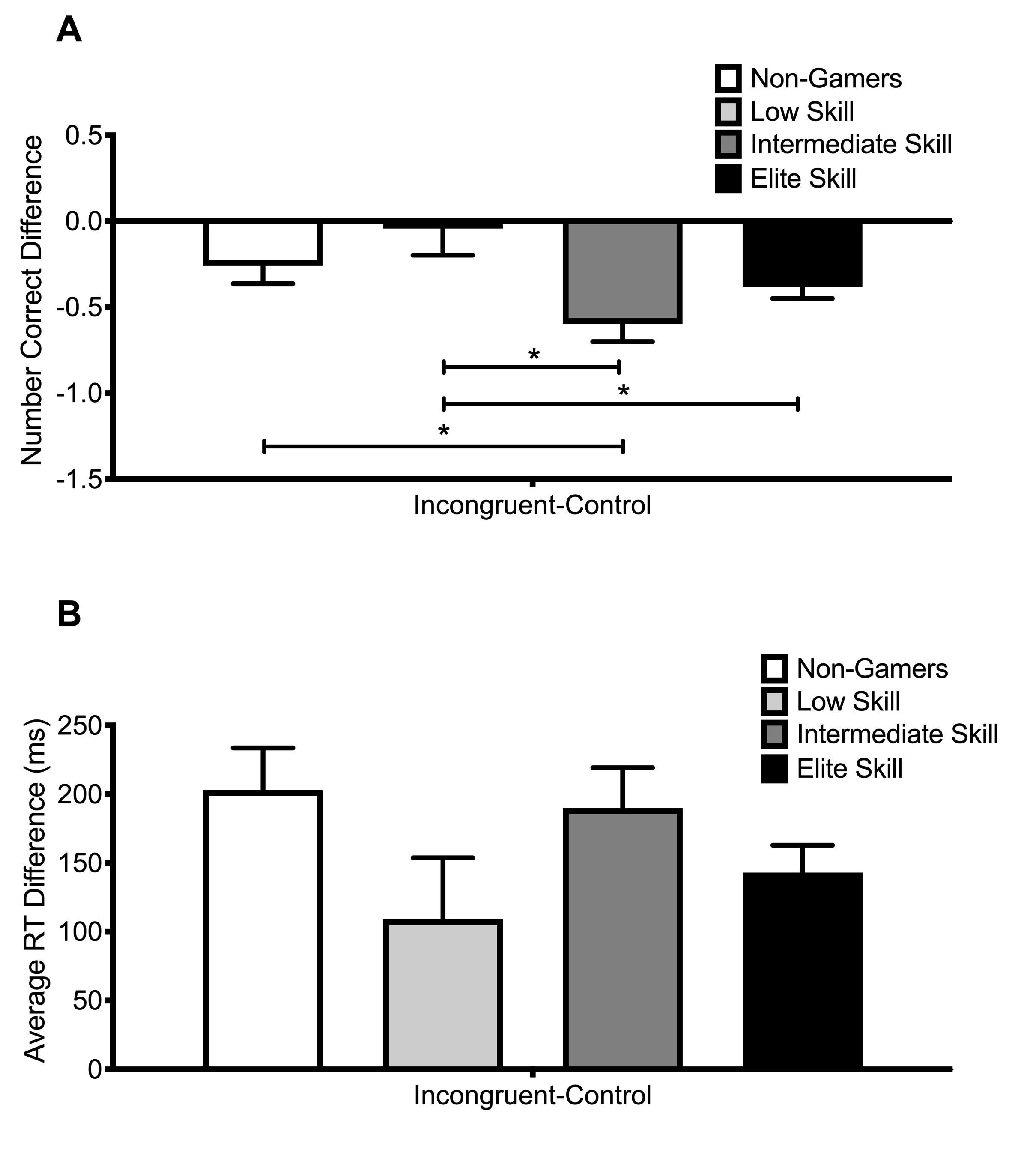

Supplement: Supplementary file 1 [file Image_1.TIFF]
